# Comparison of Misoprostol and Dinoprostone for elective induction of labour in nulliparous women at full term: A randomized prospective study

**DOI:** 10.1186/1477-7827-2-70

**Published:** 2004-09-27

**Authors:** Evangelos G Papanikolaou, Nikos Plachouras, Aikaterini Drougia, Styliani Andronikou, Christina Vlachou, Theodoros Stefos, Evangelos Paraskevaidis, Konstantinos Zikopoulos

**Affiliations:** 1 Department of Obstetrics and Gynecology, University Hospital of Ioannina, Medical School of Ioannina, Ioannina, Greece; 2 Department of Neonatology, University Hospital of Ioannina, Medical School of Ioannina, Ioannina, Greece

## Abstract

**Background:**

The objective of this randomized prospective study was to compare the efficacy of 50 mcg vaginal misoprostol and 3 mg dinoprostone, administered every nine hours for a maximum of three doses, for elective induction of labor in a specific cohort of nulliparous women with an unfavorable cervix and more than 40 weeks of gestation.

**Material and Methods:**

One hundred and sixty-three pregnant women with more than 285 days of gestation were recruited and analyzed. The main outcome measures were time from induction to delivery and incidence of vaginal delivery within 12 and 24 hours. Admission rate to the neonatal intensive care unit within 24 hours post delivery was a secondary outcome.

**Results:**

The induction-delivery interval was significantly lower in the misoprostol group than in the dinoprostone group (11.9 h vs. 15.5 h, p < 0.001). With misoprostol, more women delivered within 12 hours (57.5% vs. 32.5%, p < 0.01) and 24 hours (98.7% vs. 91.4%, p < 0.05), spontaneous rupture of the membranes occurred more frequently (38.8% vs. 20.5%, p < 0.05), there was less need for oxytocin augmentation (65.8% vs. 81.5%, p < 0.05) and fewer additional doses were required (7.5% vs. 22%, p < 0.05). Although not statistically significant, a lower Caesarean section (CS) rate was observed with misoprostol (7.5% vs. 13.3%, p > 0.05) but with the disadvantage of higher abnormal fetal heart rate (FHR) tracings (22.5% vs. 12%, p > 0.05). From the misoprostol group more neonates were admitted to the intensive neonatal unit, than from the dinoprostone group (13.5% vs. 4.8%, p > 0.05). One woman had an unexplained stillbirth following the administration of one dose of dinoprostone.

**Conclusions:**

Vaginal misoprostol, compared with dinoprostone in the regimens used, is more effective in elective inductions of labor beyond 40 weeks of gestation. Nevertheless, this is at the expense of more abnormal FHR tracings and more admissions to the neonatal unit, indicating that the faster approach is not necessarily the better approach to childbirth.

## Introduction

Induction of labor is carried out for maternal and fetal indications. One of the most common indications is prolonged pregnancy [1]. Recent studies have suggested that by continuing pregnancy beyond 41 weeks, there is a statistically significant higher perinatal morbidity and mortality as well as an increased risk to the mother [2,3]. Thus, there is a growing body of evidence suggesting the elective induction of labor at 41 weeks of gestation instead of expectant management [4-6].

Prostaglandin analogues, dinoprostone (PGE_2_) and misoprostol (PGE_1_), are widely used in "induction of labor" practice for ripening the cervix and stimulating uterine contractions in order to achieve vaginal delivery. Although dinoprostone has been approved by the FDA for cervical ripening in women at or near term, misoprostol is not currently approved for such use by the FDA, although it has the advantages of lower cost, no need for refrigeration and probably higher efficacy.

Several studies have demonstrated a higher efficacy of vaginally administered misoprostol compared to vaginal dinoprostone for both cervical ripening and labor induction [7-19]. The Cochrane Pregnancy and Childbirth Group, having reviewed 45 randomized studies, concluded that vaginal misoprostol (25 to 100 mcg) was more effective than oxytocin or dinoprostone at the usual recommended doses for induction, but with increased rates of uterine hyperstimulation both with and without associated fetal heart rate (FHR) changes, as well as meconium stained fluid [9]. Most of the studies included in the previous meta-analysis used the 50 mcg dose for misoprostol at a maximum interval of six hours between the repeated doses, always resulting in higher rates of hyperstimulation.

However, it is difficult to interpret previously published studies comparing misoprostol with dinoprostone for induction of labor since the majority of them are not double-blinded [9] and they have included both complicated and uncomplicated pregnancies, multiparous women with nulliparous as well as a wide gestational age (GA) range (37–42 weeks). Moreover, to reduce the risk of side effects, one can either decrease the dose of the drug [7,19] or prolong the dosage interval [14,15,19]. In addition, Alexander et al. have recently shown that in prolonged pregnancies it was not the induction per se that would increase the risk for caesarean section (CS), but patients related risk factors such as nulliparity and undilated cervix and the use of epidural analgesia [20].

This study was undertaken to compare the efficacy of vaginal misoprostol (50 mcg) with that of vaginal dinoprostone (3 mg) when both are administered at an interval of nine hours between repeated doses in a well-homogenized cohort of full term pregnancies (nulliparous women with an unfavorable cervix and without pregnancy complications).

## Material and Methods

Between March 1, 2001 and July 16, 2003, 163 women were recruited for the study: 80 women in the misoprostol group and 83 women in the dinoprostone group. All of the women were recruited at Ioannina University Hospital, a tertiary referral center for high-risk pregnancies, with about 1600 deliveries a year. The Ethics Committee of the University of Ioannina approved the study and all participants gave their written informed consent after they had been made aware of the purpose of the study. Although the main indication was prolonged pregnancy, some of the inductions were performed at the patient's request after consultation at 40 weeks of gestation, (without any medical indications) and only if they had not delivered by the 285^th ^day of gestation. A sequence from a computerized random number generator was used for the allocation of patients to each group. The vaginal administration of prostaglandins was performed by one of the resident doctors on duty, who was not involved in managing these women in labor or delivery. The study was double blind, since the patients were not aware of which type of medication was used, and the deliveries were then performed by two gynecologists blinded to the induction regimen utilized.

Inclusion criteria were: 1) age>18 years old 2) nulliparity, 3) accurate dating of gestation, including crown rump length (CRL) measurements in the first trimester of pregnancy, 4) singleton viable pregnancy, 5) gestational age ≥ 285 days, 6) cephalic presentation, 7) unfavorable cervical status defined as a Bishop score (BS) of ≤5, 8) intact membranes, 9) reactive non-stress test (NST). Exclusion criteria were: 1) known contraindications to receiving prostaglandins, 2) placenta previa, 3) prior uterine surgery and 4) any antenatal complications.

GA was estimated by ultrasound biometry (via CRL measurements in the first trimester of pregnancy) in cases where there were more than 3 days difference from that obtained from the last menstrual period (LMP) [21]. Uterine tachysystole was defined as more than five contractions per 10 minutes, uterine hypertonus as when one contraction lasted more than 2 minutes and hyperstimulation syndrome as the presence of non-reassuring FHR tracing combined with either tachysystole or hypertonus. Non-reassuring FHR patterns were defined as persistent or recurring episodes of severe variable decelerations, late decelerations, prolonged fetal bradycardia or a combination of decreased beat-to-beat variability and a decelerative pattern [22].

A NST to ensure the well-being of the fetus was performed for each patient at the time of recruitment and admission to the hospital (at least 285 days of gestation) and one hour before the application of the prostaglandin. After the reassessment of the cervical BS, either 50 mcg misoprostol, or 3 mg dinoprostone was administered in the posterior vaginal fornix at 23:00 hours. The NST was repeated (duration of two hours) after 1 h and 5 h. If the woman was in active labor, the membranes spontaneously ruptured or the FHR not reassuring, the patient was transferred to the labor room. Otherwise, a second BS evaluation was carried out the next morning at 08:00 am (after 9 hours). If the cervix was favorable, (BS ≥ 5), the patient was admitted to the labor ward where oxytocin augmentation was carried out if the uterine contractions were unsatisfactory and amniotomy was performed when appropriate. If the cervix was still unfavorable, a second dose of misoprostol or dinoprostone was given and the same evaluation steps as described above were followed. After a total of 18 h had elapsed, non-responders were given a third dose of prostaglandin. When the third dose was insufficient for initiating spontaneous labor, a trial of labor was offered with oxytocin infusion and if no progress was achieved within 6 hours (based on digital assessment of the BS), the patient underwent a CS.

The outcome measures were divided into "obstetrical" and "neonatal". The primary outcome measures were time from induction to delivery and incidence of vaginal delivery within 12 and 24 hours; the secondary outcomes were the CS rate, the need for oxytocin augmentation, the incidence of meconium stained amniotic fluid, the incidence of uterine tachysystole, abnormal FHR tracings, maternal morbidity, the admission to neonatal intensive care within 24 hours and neonatal arterial cord ph, base deficit.

Statistical analysis was performed using SPSS version 11 software. The Chi square test and Fisher's exact test were used to analyze nominal variables in the form of frequency tables. Normally distributed (Kolmogorov-Smirnov Test with Lilliefors correction) metric variables were tested by the T-test for independent samples, while non-normally distributed metric variables were analyzed by the Mann-Whitney U test. All tests were two-tailed with a confidence level of 95% (p < 0.05). Values are expressed as mean ± standard error (SEM).

## Results

The two groups were comparable in terms of patients' age (28.1 years vs.27.5, p > 0.05) and indication for induction (prolonged pregnancy 81.2% vs.78.3%, p > 0.05; social 18.8% vs. 21.7 %,) in the misoprostol and dinoprostone groups, respectively. Gestational age (286 days, range:285–292) and the preinduction BS (2.7 ± 0.1) in the misoprostol group were also comparable to the dinoprostone group (286 days, range:285–293) and (2.9 ± 0.1), respectively.

### Obstetrical outcome

The induction-delivery interval was significantly shorter (11.9 h vs. 15.6 h, p < 0.001) in the misoprostol group, with even less need for a second or third dose (7.5% vs. 22%, p < 0.05) compared to dinoprostone. With misoprostol, more women delivered within 12 h (57.5% vs. 32.5%, p < 0.01) and almost all of the women delivered within 24 h (98.8% vs. 91.6%, p < 0.05). In addition, spontaneous rupture of the membranes occurred more often after the administration of misoprostol (p < 0.05) and there was a reduced need for oxytocin augmentation in labor: 65.8% vs. 80.7% with dinoprostone (p < 0.05). However, uterine tachysystole (p < 0.05)) and meconium stained amniotic fluid (p > 0.05) occurred more often in the misoprostol group as did abnormal heart rate tracing (22.5% vs.12%, p > 0.05) (Table 1).

**Table 1:** Obstetrical Outcomes

	**Misoprostol n = 80 (%)**	**Dinoprostone n = 83 (%)**	**Statistical significance**
**Time from induction to delivery (h ± SEM)**	11.9 ± 0.6	15.6 ± 0.7	p < 0.001
**Delivery < 12 h**	46 (57.5%)	27 (32.5%)	p < 0.01
**Delivery < 24 h**	79 (98.8%)	76 (91.6%)	p < 0.05
**Number of doses**			
**Single dose**	74 (92.4%)	65 (78.3%)	p < 0.05
**Second dose**	6 (7.5%)	17 (20.5%)	
**Third dose**	0 (0%)	1 (1.2%)	
**Required oxytocin augmentation**	53(65.8%)	67(80.7%)	p < 0.05
**Spontaneous rupture of membranes**	31 (38.8%)	17 (20.5%)	p < 0.05
**Meconium stained AF**	15 (18.8%)	7 (9.6%)	NS
**Abnormal FHR**	18 (22.5%)	10 (12%)	NS
**Uterine Tachysystole**	10 (12.6%)	3 (3.6%)	p < 0.05
**Uterine Hyperstimulation**	2 (2.5%)	1 (1.2%)	NS

FHR = fetal heart rate.

AF = amniotic fluid

NS = not significant (p > 0.05)

SEM = standard error of the mean

In both groups, the majority of women had vaginal delivery, 92.5% with misoprostol, and 86.7% with dinoprostone. There was no statistically significant difference between the two groups with regard to the CS rate (Table 2). There were no uterine ruptures or other major maternal complications resulting from the use of either of the prostaglandins. There was only one wound infection with dinoprostone, one woman in each group had delayed discharge due to persistent pyrexia and two women in the dinoprostone group required uterine packing (insertion of tampons within the uterine cavity) due to postpartum bleeding.

**Table 2:** Mode of delivery and indications for Caesarean section

	**Misoprostol n = 80 (%)**	**Dinoprostone n = 83 (%)**	**Statistical significance**
**Vaginal**	74 (92.5%)	72 (86.7%)	NS^1^
Spontaneous vaginal	46 (57.5%)	52 (62.6%)	NS
Vacuum assisted vaginal	28 (35.0%)	20 (24.1%)	NS
**Caesarean section**	6 (7.5%)	11 (13.3%)	NS
Nonreassuring FHR^2^	4 (5.0%)	6 (7.2%)	NS
Failed induction	0 (0.0%)	1 (1.2%)	NS
Lack of labor progress	1 (1.3%)	2 (2.4%)	NS
Cephalopelvic disproportion	1 (1.3%)	2 (2.4%)	NS

^1^NS = not significant

^2^FHR = fetal heart rate.

### Neonatal outcome

More neonates in the misoprostol group had first minute Apgar scores lower than 7 (12.6% vs. 6.1%, p > 0.05), or needed neonatal resuscitation (11.4% vs. 9.9%, p > 0.05) but none of the babies had birth asphyxia [23]. The mean cord pH and the base deficit were comparable in the two groups. No neonate had meconium aspiration syndrome. Two neonates in the dinoprostone group had clavicle fracture (Table 3). There was no statistically significant difference in the number of neonates admitted to neonatal intensive care within 24 hours after delivery, between the misoprostol and dinoprostone groups (6.3% vs. 3.6% p > 0.05) (Table 4).

**Table 3:** Neonatal Outcomes

	**Misoprostol n = 80 (%)**	**Dinoprostone n = 83 (%)**	**Statistical significance**
**Birth weight (g) ^1^**	3275 ± 430	3373 ± 390	NS
**Perinatal death**	0	1(1.2%)	NS
**Neonatal resuscitation**	9 (11.3%)	9 (10.8%)	NS
**O_2 _Supplementation**	1	2	
**Ambou ventilation**	7	6	
**Intubation in labor room**	1	1	
**Apgar score < 7**			
**1 min**	10 (12.5%)	5 (6.0%)	NS
**5 min**	1 (1.3%)	0	
**Cord blood pH (arterial)^1^**	7.28 ± 0.05	7.27 ± 0.05	NS
**Base deficit ^1^**	5.0 ± 2.3	5.7 ± 3.2	NS
**7.01 < cord pH < 7.20**	3 (3.8%)	4 (4.8%)	NS
**10 < base deficit < 16**	1 (1.3%)	2 (2.5%)	NS
**Hyperbilirubinemia ^2^**	9 (11.3%)	5 (6.0%)	NS
**Birth trauma ^3^**	0	2 (2.5%)	NS

^1^Values expressed as mean ± SD

^2^Excluding pathological causes of icterus

^3^Both were clavicle fractures

**Table 4:** Admission to Neonatal Intensive Care Unit

**N (%)**	**DA^1^**	**Delivery^2^**	**Indication**	**Diagnosis**	**HDs^3^**
**Within 24 hours**					

**Misoprostol† 5 (6.3%)**	01	VVD	Rule out infection	Elevated CRP^4^WBC^5^	07
	01	VVD	Respiratory distress	Respiratory infection	11
	01	CS	Rule out asphyxia	Infection	10
	01	SVD	Respiratory distress	Work up for infection	03
	01	VVD	Respiratory distress	Work up for infection	04
**Dinoprostone† 3 (3.6%)**	01	VVD	Respiratory distress	Atelectasis	06
	01	SVD	Rule out asphyxia	Infection	10
	01	SVD	Respiratory distress	Respiratory infection	10
**After 24 hours**					

**Misoprostol† 6 (7.2%)**	02	VVD	Rule out infection	WBC in CSF^6^	10
	07	VVD	Hyperbilirubinemia	Urinary infection	07
	04	SVD	Hyperbilirubinemia	Icterus	04
	08	SVD	Infection	Respiratory infection	10
	03	SVD	Feeding difficulty	WBC in CSF	20
	05	CS	Hyperbilirubinemia	Icterus	04
**Dinoprostone† 1 (1.2%)**	17	SVD	Fever	WBC in CSF	15

^1 ^DA = day of admittance

^2 ^SVD = Spontaneous Vaginal Delivery, VVD = vacuum assisted vaginal delivery, CS = Caesarean section

^3 ^HDs = hospitalization days

^4 ^CRP = C-reactive protein

^5 ^WBC = white blood cell

^6 ^CSF = cerebrospinal fluid

† not significant (p > 0.05)

A 28-year-old woman at 41 weeks of gestation had an unexplained stillbirth after receiving a single dose of dinoprostone. Seven hours later she had a cardiotocogram without abnormal FHR patterns and regular contractions of the uterus were evident. We decided to move her to the labor ward and within half an hour of entry, no cardiac activity of the fetus was found. During these 30 minutes FHR monitoring had been discontinued, as it was not included in the study design. We decided to let her attempt vaginal delivery. An amniotomy was performed and the amniotic fluid was found to be clear and a vaginal delivery was achieved within 6 hours. Direct examination of the fetus, the placenta and the umbilical cord (UC) showed only a thin UC with excess twisting around its axis. The anatomopathology examination of the fetus revealed no abnormality except a microscopically decreased Wharton's jelly.

## Discussion

Nowadays, induction of labor is more widely used than ever before [24,25]. Recent studies have shown that this increase is mainly due to a rise of inductions for marginal or elective reasons. The common indications are elective induction and postdate pregnancy often applied to gestations of 40 to 41 weeks [1,25]. Mongelli et al. have also shown that for the detection of post-maturity there is no advantage in using menstrual dates when ultrasound biometry is available [26]. Women may experience distress when labor has not started by the expected date [27] and obstetricians have to withstand pressure from these patients as well as the temptation to use prostaglandins earlier. Appropriate evaluation of the pregnancy and consultation with such patients will lead to the correct selection of those who will benefit most from a labor induction, thus eliminating the risk of post-maturity to the fetus without inducing fetal distress during labor.

To the best of our knowledge, the present study is the only one that compares misoprostol and dinoprostone in such well-homogenized groups. All of the women were nulliparous with intact membranes and at more than forty weeks' gestation with no antenatal complications and all had an unfavorable cervix. In these carefully selected patients, misoprostol at the dose used not only shortened the time between induction and delivery (11.9 vs. 15.6 h), but it also was significantly more effective than dinoprostone. The positive point was that this result was achieved with a very low CS rate even in the dinoprostone group, (7.5%, and 13.3%), respectively. A difference of 5% in favor of misoprostol, although not statistically significant, might have clinical importance in terms of patient health and cost effectiveness. Although in the recent large meta-analysis [9] published by the Cochrane Library, the CS rates were inconsistent, they tended to be lower with misoprostol; an earlier study by Sanchez-Ramos et al. found a statistically significant difference in favor of misoprostol [28]. In addition, our results for this GA window are reassuring with regard to concerns that have been raised from previous retrospective studies reporting an increased risk of Caesarean delivery in nulliparous women when elective inductions are performed [29,30].

Even though misoprostol improves the kinetics of labor during induction in a more efficient way than dinoprostone, concerns persist with respect to intrapartum fetal "wellbeing". In order to avoid uterine hyperstimulation and abnormal FHR tracings, we used for first time in the literature, a 9 h interval between the prostaglandin doses. Although we indeed achieved a low rate of uterine hyperstimulation syndrome (2.5% with misoprostol and 1.2% with dinoprostone, respectively), we still noticed a trend towards a high rate of abnormal FHR tracings during induction with misoprostol. Our findings, in accordance with the previous Cochrane metanalysis [9], showed that with misoprostol there was an increased probability of meconium staining of amniotic fluid as well as of uterine tachysystole and of abnormal FHR tracings. In the misoprostol group, the majority of women also underwent either a CS or a vacuum operative delivery due to non-reassuring FHR. If neonatal outcomes such as neonatal resuscitation, low Apgar score in the first minute and admittance to the neonatal unit within the first 24 hours (none of the above were statistically significant but they were more frequent with misoprostol) are taken into account, misoprostol may increase these complications in labor. Thus, although our sample size cannot determine safety, misoprostol use is associated with a higher chance of admittance to the neonatal unit within 24 hours even in the absence of asphyxia. This evidence indicates that the faster approach to childbirth is not necessarily the better one.

Attempting an explanation to the aforementioned side effects of misoprostol use and taking into account other reports [9,31,32], it appears that the increase in clinically relevant adverse effects is not only misoprostol related but it may be dose dependent. Lyons et al. have recently shown in term pregnant rats that a higher dose of misoprostol is needed to induce PGE2 secretion in the cervix than in the myometrium, and furthermore that EP3 receptors (prostaglandin E2 receptors) are differentially expressed in the myometrium (increased) than in the cervix (unaltered) in response to misoprostol [33]. The above findings indicate that misoprostol not only acts better on the myometrium than on the cervix, but an even higher dose is needed in order to ripen the cervix. Thus, it seems reasonable that increasing the interval between repeated misoprostol doses should reduce the risk of an asynchrony between a well or even hyper-stimulated uterus and a still not efficiently ripened cervix. Misoprostol probably has a large inter-patient variability in terms of pharmacokinetics, but it is also probable that the 50 mcg dosage may induce asynchrony between immature cervix effacement and uterine contractions, resulting in a more rapid but also more "stressful" labor. Based on these findings, we would propose, in future, a slight modification of the misoprostol protocol used in this study. An initial lower dose of misoprostol (20–25 mcg), followed by 50 mcg should be considered in trying to achieve priming of the cervix without inducing such high uterine contractility and neonatal complications. Indeed, in a recent study comparing 25 mcg misoprostol with 1 mg dinoprostone administered vaginally every four hours, the admission rate to neonatal intensive unit was significantly lower in the misoprostol group [34].

It still has to be mentioned that in many of our participants, the vertex was not engaged in the pelvic inlet on the day of admittance and this should have been included as an independent risk factor in the initial study design. The exact cause of the stillbirth in the dinoprostone group remains unclear, emphasizing thus, the need for continuous FHR monitoring during labor induction if regular uterine contractions persist [35,36].

## Conclusions

To conclude, 50 mcg misoprostol at a 9 h interval is more highly effective in promoting cervical ripening and in inducing labor, compared to dinoprostone. However, certain aspects concerning fetal well being during labor induction remain questionable. Larger prospective studies comparing elective induction to expectant management after a completed 40-week gestation (on the basis of early ultrasound biometry) might reveal a subgroup of women, such as nulliparous with an unfavorable cervix, who might benefit from an elective induction, preferably with a 25 mcg misoprostol initial dose.

## Authors' contributions

**E.P: **conceived of the study, participated in the sequence alignment, performed the statistical analysis and drafted the manuscript.

**N.P: **conceived of the study, and performed the labor inductions

**A.D: **performed the neonatal examination and follow up

**S.A: **performed the neonatal examination and participated in the neonatal data analysis

**C.V: **was the midwife involved in women allocation, medications preparation and labour data registration

**T.S: **conceived of the study and data analysis

**E.P: **conceived of the study and coordinated the study

**K.Z: **conceived of the study, performed the labor inductions and coordinated the study
